# Serum creatinine and estimated glomerular filtration rates in HIV positive and negative adults in Ethiopia

**DOI:** 10.1371/journal.pone.0211630

**Published:** 2019-02-12

**Authors:** Daniel Yilma, Alemseged Abdissa, Pernille Kæstel, Markos Tesfaye, Mette F. Olsen, Tsinuel Girma, Christian Ritz, Henrik Friis, Åse B. Andersen, Ole Kirk

**Affiliations:** 1 Department of Internal Medicine, Jimma University, Jimma, Ethiopia; 2 Department of Laboratory Sciences and Pathology, Jimma University, Jimma, Ethiopia; 3 Department of Nutrition, Exercise and Sports, University of Copenhagen, Copenhagen, Denmark; 4 Department of Psychiatry, St. Paul’s Hospital Millennium Medical College, Addis Ababa, Ethiopia; 5 Department of Paediatric and Child Health, Jimma University, Jimma, Ethiopia; 6 Department of Infectious Diseases, Rigshospitalet, Copenhagen and Research Unit of Infectious Diseases, Department of Clinical Research, University of Southern Denmark, Denmark; University of Colorado Denver School of Medicine, UNITED STATES

## Abstract

**Background:**

Glomerular filtration rate estimating equations using serum creatinine are not validated in most African settings. We compared serum creatinine and estimated glomerular filtration rate (eGFR) in HIV positive and negative adults and assessed the performance of eGFR equations ((Cockcroft and Gault (CG), Modification of Diet in Renal Disease (MDRD), and Chronic Kidney Disease Epidemiology Collaboration (CKD-EPI)) compared to 24-hour creatinine clearance in HIV positive adults.

**Methods:**

Data were collected on demographic, anthropometric, body composition, clinical parameters and serum creatinine in HIV positive and negative adults. 24-hour urine was collected from some of the HIV positive adults who volunteered. Bias was calculated as mean difference between 24-hr creatinine clearance and eGFR (eGFR– 24 hour creatinine clearance) and the accuracy of each eGFR equation was calculated as the percentage of estimates within 30% of creatinine clearance.

**Results:**

A total of 340 HIV positive and 100 HIV negative adults were included in this study. Creatinine clearance was determined for 46 of HIV positive adults. Serum creatinine increased with increasing age, weight, height, body surface area, fat free mass and grip strength in both HIV positive and negative adults (P<0.05). No difference was observed in eGFR between HIV positive and HIV negative adults. For all eGFR equations, the correlation between eGFR and 24-hr creatinine clearance was 0.45–0.53 and the accuracy within 30% of 24-hr creatinine clearance was 24–46%. Removing ethnic coefficient reduced the bias and improved accuracy of the CKD-EPI and the MDRD estimates.

**Conclusion:**

Ethiopian HIV positive adults in the present study had good kidney function at the initiation of antiretroviral treatment. However, all eGFR equations overestimated 24-hr creatinine clearance in the study population. Creatinine based eGFR equations that accounts for low muscle mass and body surface area are needed.

## Introduction

HIV infection directly or indirectly affects the kidneys and result in HIV-associated nephropathy (HIVAN), immune complex kidney disease and thrombotic microangiopathy [1]. Moreover, HIV patients can develop renal impairment due to long-term exposure to antiretroviral treatment (ART) or comorbid illnesses [2].

Kidney function is assessed by determination of glomerular filtration rate (GFR). Precise assessment of GFR is performed using ^125^I-iothalamate or ^51^Cr-EDTA measurements [3,4]. 24-hour creatinine clearance also provides a good estimate of GFR, especially in patients with low lean body mass [5]. However, these GFR determination procedures are either costly or require 24-hour urine collection and therefore not commonly used in routine clinical practice. To overcome this problem, several serum creatinine-based GFR estimating equations have been developed in the past. Cockcroft and Gault (CG) [6], the Modification of Diet in Renal Disease (MDRD) [7], and the Chronic Kidney Disease Epidemiology Collaboration (CKD-EPI) [8], have proposed GFR estimating equations that are widely used in clinical practice. These equations uses different variables as serum pool of creatinine can be affected by multiple factors like age, muscle mass and diet [9].

HIV patients in many developing countries are often initiated on ART without baseline GFR determination, as serum creatinine measurement tools are not easily accessible. Previous studies in Africa have shown variable burden of renal disease in HIV patients [10–13]. However, few studies compared the renal function of ART naïve HIV positive adults with HIV negative adults. Besides, there are limited studies that have validated GFR estimating equations in HIV positive adults in Africa. Therefore, the aim of the present study was to compare serum creatinine and estimated glomerular filtration rate (eGFR) in HIV positive and negative adults and also to compare eGFR equations with 24-hour creatinine clearance in HIV positive adults.

## Materials and methods

### Study design and setting

This study used the data from a nutritional intervention trial that was conducted in Jimma, southwest Ethiopia. The details of the trial design and methods of recruitment have been described elsewhere [14]. The trial was registered at www.controlled-trials.com (ISRCTN32453477) and www.pactr.org (PACTR201110000330271).

### Participants

**Fig 1:**
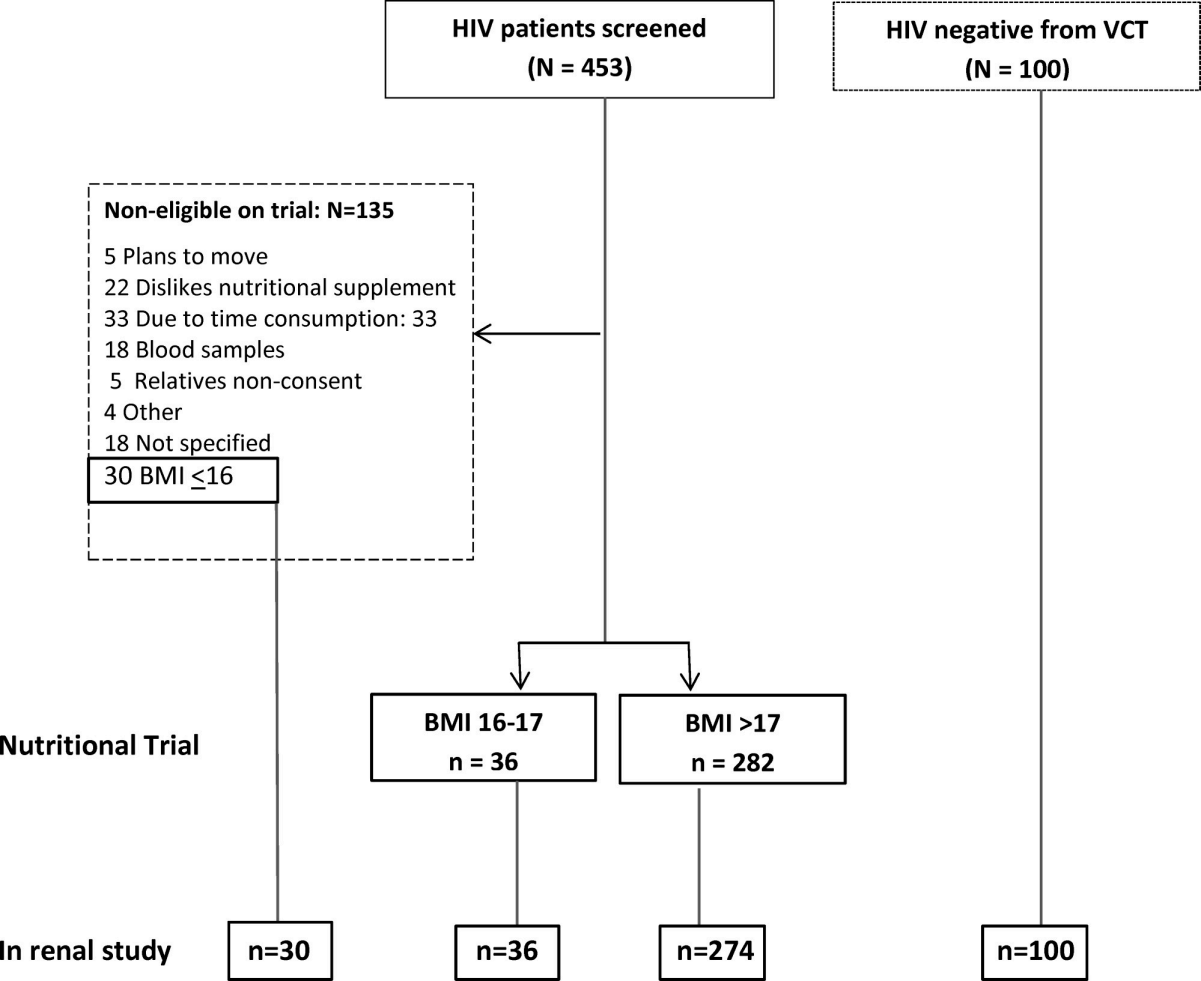
Flow chart showing participants included on this renal study from nutritional intervention trial.

HIV positive adults who were eligible for ART at Jimma University Specialized Hospital (JUSH), Jimma Health Centre (JHC) and Agaro Health Centre (AHC) were invited to participate in the nutritional trial [14]. ART eligibility criteria were CD4 count < 200 or CD4 count < 350 and WHO stage 3 or 4 disease or WHO stage 4 diseases without CD4 count determination. HIV positive adults who were ≥18 years of age, not pregnant or lactating, BMI>16 kg/m^2^ with no current use of nutritional supplements and living within 50 km of the recruitment sites were enrolled to the trial. Thirty HIV positive adults who were not included in the nutritional trial due to low BMI were included in this study. The HIV negative reference group matched for sex, age (±3 years) and BMI group to the last 100 included HIV positive adults and no known acute or chronic diseases, were recruited among confirmed HIV negative adults from the voluntary counseling and testing service at JUSH (Fig 1).

### Demographic and clinical data

Demographic data were collected by trained study nurses who used structured questionnaires in the local languages Amharic or Afaan Oromo. Clinical data were collected by health professionals working in the ART clinics.

### Body composition

Weight and height were measured with calibrated scales and stadiometers, respectively, with the participant barefoot and wearing minimal clothing and body mass index (BMI) was calculated as weight (kg)/height (m)^2^. Body composition in HIV positive adults was assessed using the deuterium dilution method. A 30 g deuterium oxide (99.8% ^2^H, Sercon, Crewe, UK) weighed with 0.01 g precision was given orally after collection of pre-dose saliva samples [15]. Post-dose saliva samples were collected after four hours’ equilibration. Saliva enrichment of deuterium was determined by Fourier Transform Infrared Spectrometer (IRAffinity-1, Shimadzu, Kyoto, Japan). Total body water was calculated from post-dose deuterium enrichment with adjustment for pre-dose enrichment, using a factor of 1.041 to adjust for proton exchange. Lean body mass was calculated based on an assumed hydration factor of 73.2% [15]. Body composition in HIV negative adults was measured with air displacement plethysmography (BodPod, USA).

### Laboratory

The study laboratory personnel collected 10 ml of fasting venous blood in an EDTA tube, and 10 ml in a plain tube. One ml of whole blood from EDTA blood collection tube was transferred to small tubes and CD4+ T cells were enumerated using the Facscount (Becton-Dickinson, New Jersey, USA). After centrifugation, serum or plasma was separated and transferred to cryotubes in aliquots of 1 ml and stored at -80°C. Plasma was shipped to the International Clinical Laboratory in Addis Ababa, Ethiopia, for viral load quantification and serum to University of Copenhagen, Denmark, for analysis of C-reactive protein (CRP) and creatinine.

Participants who volunteered from the HIV positive adults group to collect 24-hour urine were provided with urine collecting container and instruction. Participants were asked to return with the urine container after 24 hours and a blood sample for serum creatinine determination was taken. We used 24 hours urine collected for creatinine clearance measurement if the participant witnessed s/he collected as per instruction provided, total urine volume was >400 ml/24hr and urine creatinine was > 20 mg/dl (not diluted).

Creatinine was measured using a colorimetric kinetic assay (HORIBA ABX A11A01933) for Pentra 400 (HORIBA ABC, Montpellier, France). The results are given in μmol/L and the precision of the assay was 6.5 CV% based on repeated measurements of a normal serum in each run (mean ± SD: 80.7 ± 5.2 μmol/L).

HIV-1 load was quantified using a commercial PCR assay (RealTime HIV-1, Abbott Laboratories, Illinois, USA) using automated extraction system (m2000 Real Time System, Abbott Laboratories, Illinois, USA). Virus RNA was amplified on the m2000rt platform (Abbott Laboratories, Illinois, USA). C-reactive protein (CRP) was measured in serum using an immunoturbidimetric assay (HORIBA ABX A11A01611) for Pentra 400 (HORIBA ABC, Montpellier, France). The results are given in mg/L and the precision of the assay was 8.3 CV% based on repeated measurements of a normal serum in each run (mean ± SD: 0.71 ± 0.06 mg/L).

### Glomerular filtration estimates

GFR was estimated using CG [6], four variable MDRD [7] and CKDEPI [8] equations (Box 1).

**Box 1. Glomerular filtration rate estimating equations**

Males:

eGFR_CG_ = (140 − age) × body weight/serum creatinine × 72

eGFR_MDRD_ = 175 × serum creatinine^−1.154^ × age^−0.203^

eGFR_MDRD in blacks_ = 175 × serum creatinine^−1.154^ × age^−0.203^ × 1.21

eGFR_CKD-EPI_ if serum creatinine ≤0.9 μmol/L:

    eGFR_CKD-EPI in non-blacks_ = (serum creatinine/0.9)^−0.411^ × 0.993^age^ × 141

    eGFR_CKD-EPI in blacks_ = (serum creatinine/0.9)^−0.411^ × 0.993^age^ × 163

eGFR_CKD-EPI_ if serum creatinine >0.9 μmol/L:

    eGFR_CKD-EPI in non-blacks_ = (serum creatinine/0.9)^−1.209^ × 0.993^age^ ×144

    eGFR_CKD-EPI in blacks_ = (serum creatinine/0.9)^−1.209^ × 0.993^age^ ×166

Females:

eGFR_CG_ = (140 − age) × body weight/serum creatinine × 72 × 0.85

eGFR_MDRD_ = 175 × serum creatinine^−1.154^ × age^−0.203^ × 0.742

eGFR_MDRD in blacks_ = 175 × serum creatinine^−1.154^ × age^−0.203^ × 0.742 × 1.21

eGFR_CKD-EPI_ if serum creatinine ≤0.7 μmol/L:

    eGFR_CKD-EPI in non-blacks_ = (serum creatinine/0.7)^−0.329^ × 0.993^age^ × 144

    eGFR_CKD-EPI in blacks_ = (serum creatinine/0.7)^−0.329^ × 0.993^age^ × 166

eGFR_CKD-EPI_ if serum creatinine >0.7 μmol/L:

    eGFR_CKD-EPI in non-blacks_ = (serum creatinine/0.7)^−1.209^ × 0.993^age^ × 144

    eGFR_CKD-EPI in blacks_ = (serum creatinine/0.7)^−1.209^ × 0.993^age^ × 166

24-hr creatinine clearance was calculated as; CrCl = (urine creatinine mg/dl x volume of urine ml /24 hr)/ (serum creatinine mg/dl x 24 hr x 60 min/hr). Cockcroft-Gault equation and 24-hour creatinine clearance were adjusted for body surface area (BSA) of 1.73 m^2^ using the DuBois method: BSA (m^2^) = [71.84 x weight (kg) ^0.425^ x height (cm)^0.725^]/10 000 to allow comparisons with other estimates [16]. We calculated MDRD and CKDEPI equations with and without black ethnicity correction.

### Statistical analysis

Frequencies and medians (interquartile ranges [IQR]) were used for descriptive statistics. Demographic and clinical characteristics were compared between HIV positive and negative individuals using Wilcoxon rank-sum test or Pearson chi square test, as appropriate. Linear regression was used to investigate the relationships between serum creatinine with demographic, anthropometric, body composition and clinical parameters. Pearson's correlation coefficient was used to show the correlation between 24-hr creatinine clearance and eGFR equations. Bias was calculated mean difference between 24-hr creatinine clearance and eGFR using the specific eGFR equations (eGFR– 24 hr creatinine clearance) and the accuracy of each equation was calculated as the percentage of estimates within 30% of creatinine clearance [17]. Analysis of variance was used to further evaluate differences in bias between groups. Bland-Altman plots were used to explore the relationship between 24-hr creatinine clearance and eGFR using the specific equations [18]. Stata version 11.2 (StataCorp, Texas, USA) was used for all analyses.

### Ethical considerations

Written informed consent was obtained from all participants. Ethical permission was obtained from the Ethiopian National Health Research Ethical Review Committee and Jimma University Ethical Review committee. A consultative approval was obtained from the Danish National Committee on Biomedical Research Ethics. Participants were reimbursed for transport cost when they visited the clinics for the study purpose.

## Results

A total of 448 individuals, i.e. 348 HIV positive and 100 HIV negative, were recruited. Renal function test results were available for 340 HIV positive and for all HIV negative adults. HIV positive adults were older and had lower weight, body surface area, BMI and grip strength compared to HIV negative persons. The median (IQR) CD4 count of HIV positive adults was 182 (114; 242) cells/μl with 22% of participants having a CD4 count ≤100 cells/μl and 38% between 100 and 200 cells/μl. About 41% of participants had WHO stage III and IV disease with 21% (71) active opportunistic infections on treatment during the screening and 20% with previous history of opportunistic infections. Half of the patients with a recent history of an opportunistic condition were on anti-tuberculosis treatment. The other treatments that were taken by the other half were fluconazole, amoxicillin with or without clavulanate, metronidazole and cotrimoxazole for different opportunistic infections. Hepatitis B co-infection was detected in 3.9% of the HIV positive adults (Table 1). None of the participants had known history of diabetes mellitus and chronic kidney disease, but three of participants reported history of hypertension. Hepatitis C coinfection was detected in 0.6% (2/321) patients.

**Table 1:** Baseline characteristics of 340 HIV positive and 100 HIV negative adults in Ethiopia ^1^.

	HIV positive	HIV negative	P
Age, y	30 (27; 38)	29 (23; 37)	0.03
Gender			0.46
Males	112 (32.9)	29 (29.0)	
Females	228 (67.1)	71 (71.0)	
Weight, kg	48.5 (43.8; 53.9)	53.5 (47.3; 60.9)	<0.001
Height, m	1.59 (1.54; 1.66)	1.58 (1.53; 1.65)	0.71
Body surface area, m^2^	1.48 (1.39; 1.58)	1.55 (1.44; 1.65)	<0.001
Body mass index, kg/m^2^	18.8 (17.4; 20.6)	20.5 (18.6; 23.6)	<0.001
Grip strength, kg	21.7 (18.7; 26.6)	26.5 (22.4; 31.5)	<0.001
C-reactive protein, mg/L	2.0 (0.5; 7.5)	0.5 (0.2; 2.5)	<0.001
Creatinine, mg/dl	0.7 (0.5; 0.8)	0.6 (0.5; 0.7)	0.28
WHO Stage			
I	99 (29.0)	-	-
II	102 (29.9)	-	-
III	111 (32.6)	-	-
IV	29 (8.5)	-	-
CD4 count, cells/ul	182 (114; 242)	-	-
Hepatitis B surface antigen positive	13 (3.9)		
Viral load, log (copies+1/mL)	4.8 (4.3; 5.3)	-	-

^1^ Data are expressed as median (interquartile range) or number (%)

Factors that were associated with serum creatinine were similar in HIV positive and negative adults. Females had lower serum creatinine than males, and serum creatinine increased with increasing age, weight, height, body surface area, fat free mass and grip strength. In HIV positive adults except that participants with WHO stage IV disease had higher serum creatinine compared to participants with WHO stage I disease, no association was found between serum creatinine and other clinical parameters (C-reactive protein, viral load, TB treatment and hepatitis B infection) (Table 2).

**Table 2:** Factors associated with serum creatinine in 340 antiretroviral naïve HIV positive and 100 HIV negative adults in Ethiopia. Values are from simple regression analysis with coefficient B, 95% confidence interval (CI) and P value.

	HIV positive (n = 340)		HIV negative (n = 100)	
	B (95% CI)	P	B (95% CI)	P
Age, Y	0.009 (0.005; 0.012)	<0.001	0.006 (0.003; 0.008)	<0.001
Gender, female	-0.146 (-0.210; -0.0825)	<0.001	-0.159 (-0.205; -0.114)	<0.001
Weight, kg	0.004 (0.00; 0.078)	0.05	0.003 (0.001; 0.006)	0.02
Height, cm	0.005 (0.002; 0.009)	0.004	0.004 (0.001; 0.007)	0.005
Fat mass, kg	-0.002 (-0.008; 0.004)	0.50	-0.001 (-0.005; 0.002)	0.48
Fat free mass, kg	0.007 (0.002; 0.011)	0.005	0.007 (0.004; 0.011)	<0.001
Body mass index, kg/m^2^	-0.001 (-0.012; 0.011)	0.92	0.003 (-0.004; 0.009)	0.39
Body surface area, m^2^	0.282 (0.069; 0.495)	0.01	0.268 (0.102; 0.434)	0.002
Grip strength, kg	0.007 (0.002; 0.012)	0.004	0.007 (0.005; 0.011)	<0.001
C-reactive protein, mg/L	0.001 (-0.0003; 0.002)	0.17	-	-
Viral load, log (copies+1/ml)	-0.005 (-0.037; 0.026)	0.72	-	-
Hepatitis B surface antigen positive	-0.008 (-0.172; 0.155)	0.92	-	-
Tuberculosis treatment, yes	-0.053 (-0.154; 0.049)	0.31	-	-
WHO Stage				
I	Reference	-		
II	0.004 (-0.076; 0.084)	0.93		
III	-0.077 (0.079; 0.001)	0.98		
IV	0.155 (0.034; 0.276)	0.01		

No difference was observed in eGFR between HIV positive adults who were eligible for ART and HIV negative adults when using GFR estimating equations with adjustment for body surface area. HIV negative adults had higher eGFR than HIV positive adults with borderline significance when CG without body surface area adjustment was used (P = 0.056). The correlation between eGFR equations was 0.79 to 0.96. MDRD equation without ethnic factor correction showed the highest number of HIV patients with eGFR <90 mL/min/1.73 m^2^ (Table 3). GFR< 60 mL/min/1.73 m^2^ was estimated in 0.3–5% of the 340 HIV positive adults using the different eGFR equations; with higher proportions when using CG without body surface area adjustment.

**Table 3:** Estimated glomerular filtration rate (eGFR) using creatinine based equations of 340 antiretroviral naïve HIV positive and 100 HIV negative adults in Ethiopia ^1^.

	HIV positive (n = 340)		HIV negative (n = 100)		
	Median (IQR)^3^	< 90 n (%)	Median (IQR)^3^	< 90 n (%)	P^4^
CG ^2^	99.5 (83.4; 122.4)	127 (37.4)	115.1 (97.6; 133.3)	13 (13)	0.056
CG-BSA ^2^	115.2 (96.1; 138.5)	54 (15.9)	130.7 (113.1; 145.2)	6 (6)	0.41
MDRD ^2^	139.7 (115.3; 170.1)	18 (5.3)	146.3 (132.1; 161.9)	2 (2)	0.40
MDRD ^2^ without ethnic factor	115.3 (95.1; 140.4)	61 (17.9)	120.7 (109.0; 133.6)	8 (8)	0.40
CKD-EPI ^2^	138.6 (124.5; 149.1)	12 (3.5)	142.9 (134.1; 149.4)	1 (1)	0.18
CKD-EPI ^2^ without ethnic factor	119.9 (107.7; 129.1)	24 (7.1)	123.8 (116.3; 129.6)	2 (2)	0.18

^1^ Data are median (interquartile range) or number (%)

^2^ CG–Cockcroft-Gault; CG-BSA–Cockcroft-Gault adjusted for body surface area of 1.73m2; CKD-EPI–Chronic Kidney Disease Epidemiology Collaboration; MDRD–Modification of Diet in Renal Disease

^3^ Expressed as mL/min/1.73 m2, except for CG expressed as mL/min

^4^ P-value indicated is comparisons in each eGFR between HIV positive and negatives

**Fig 2:**
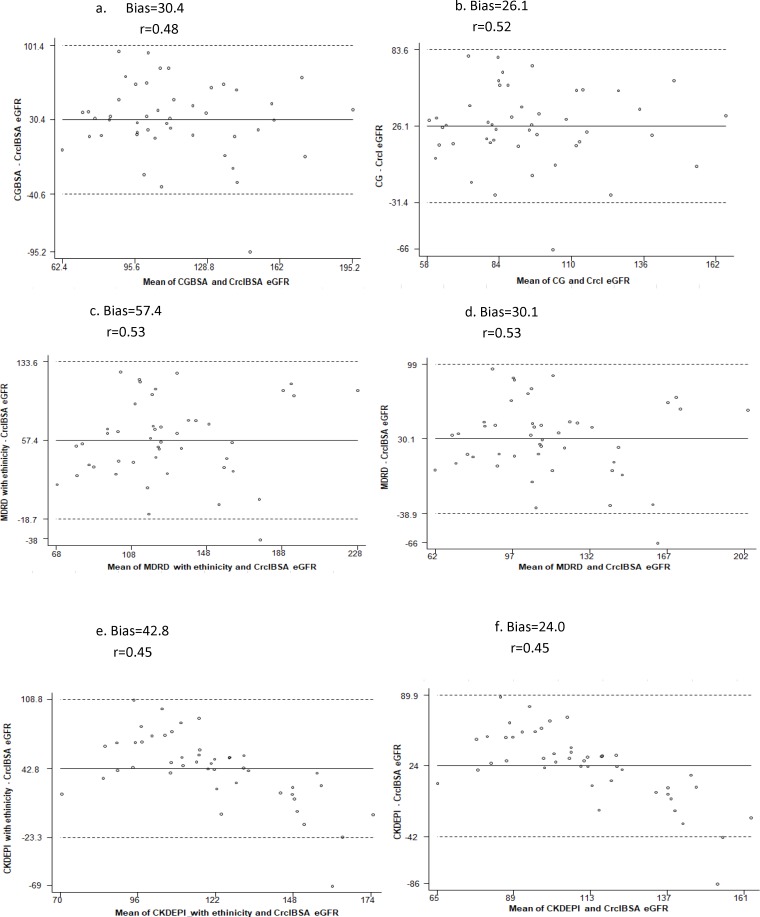
Bland Altman plots showing the agreement between creatinine clearances and estimated glomerular filtration rate with bias and correlation coefficient (r). The solid horizontal line represents the overall mean of the differences, and the dashed lines show the range containing the mean of the differences ±1.96 standard deviations, which are referred as limits of agreement. a and b. Cockcroft and Gault (CG) Estimated glomerular filtration rate (eGFR) with 24-hour creatinine clearance with and without body surface area (BSA) adjustment, c and d. Modification of Diet in Renal Disease (MDRD) eGFR without and with ethinicity, e and f. Chronic Kidney Disease Epidemiology Collaboration (CKD-EPI) without and with ethinicity.

A 24-hour urine sample was collected for 51 HIV positive adults to determine 24-hour creatinine clearance and 46 of them had appropriately collected the samples. The median (IQR) age of participants who had 24-hr creatinine clearance measurements was 29 (25, 35) years and 89.1% were female, 54.3% had BMI <18.5 and 8.7% had WHO Stage IV disease. For all eGFR equations, the correlation between eGFR and 24-hr creatinine clearance was 0.45–0.53 and the accuracy within 30% of 24-hr creatinine clearance was 24–46%. The mean eGFR_MDRD_ and eGFR_CKD-EPI_ with ethnic factor corrections was higher, but MDRD and CKD-EPI equations with ethnic factor corrections had the highest bias and lowest accuracy. Removing the ethnic coefficient reduced the bias and improved accuracy of the CKD-EPI and the MDRD estimates. Moreover, the bias decreased and correlation coefficient increased for the CG when CG and creatinine clearance were compared without body surface area adjustment. Taken together, the CKD-EPI equation without ethnic factor correction had the highest accuracy and lowest bias. During stratification of creatinine clearance as < and ≥ 90 mL/min/1.73 m^2^, the bias was lower and the accuracy within 30% of creatinine clearance was higher for all equations in participants with creatinine clearance ≥90 mL/min/1.73 m^2^ compared to participants with creatinine clearance < 90 mL/min/1.73 m^2^. However, we did not find significant difference in bias when we stratify with BMI < and ≥18.5 kg/m^2^ (Table 4 and Fig 2).

**Table 4:** Performance of creatinine-based eGFRs compared to 24 hour urine creatinine clearance (crcl) in 46 HIV positive adults in Ethiopia.

	eGFR^1^ Mean (+SD)	r^3^	Overall (n = 46)		crcl < 90 (n = 22)		crcl ≥ 90 (n = 24)	
			Bias^4^	P30^5^	Bias^4^	P30^5^	Bias^4^	P30^5^
Crcl	99.0 (37.0)	-	-	-	-	-	-	-
CG-BSA^2^	129.4 (32.8)	0.48	30.4	43.5	44.9	22.7	17.2	62.5
MDRD^2^	156.4 (34.2)	0.53	57.4	23.9	68.8	9.1	16.6	37.5
MDRD without ethnic factor^2^	129.1 (41.5)	0.53	30.1	43.4	44.7	27.3	47.0	58.3
CKD-EPI^2^	141.8 (18.4)	0.45	42.8	30.4	63.6	4.6	23.7	54.2
CKD-EPI without ethnic factor^2^	123.0 (16.0)	0.45	24.0	45.6	46.0	13.6	3.7	75.0

^1^Expressed as mL/min per 1.73 m2

^2^CG-BSA–Cockcroft-Gault adjusted for body surface area of 1.73m2; CKD-EPI -Chronic Kidney Disease Epidemiology Collaboration; MDRD-Modification of Diet in Renal Disease; eGFR- estimated glomerular filtration rate

^3^Pearson correlation coefficient

^4^The difference of means (eGFR-Crcl)

^5^The percentage of calculated values using creatinine based eGFR equations within 30% of the crcl value

## Discussion

Glomerular filtration rate of ART-naïve HIV positive adults were not different from HIV negative adults in the study population. However, commonly applied GFR estimating equations seemed to overestimate the creatinine clearance in this study population.

Serum creatinine is used as an index of renal function although it is affected by age, gender, muscle mass, liver disease and diet [9,19,20]. Our data showed and confirmed the association of serum creatinine levels with age, gender, weight, fat free mass, body surface area and grip strength in HIV positive and negative adults. Most of the associations can be explained by the impact of lean body mass on creatinine production rate. No difference in serum creatinine level was found between HIV positive and negative adults. However, HIV-positive adults had lower weight, body surface area, BMI and grip strength compared to HIV negative adults. We did not compare the fat free mass and fat mass between HIV positive and HIV negative adults as body composition assessment was performed using different methods for HIV positive and negative adults (deuterium dilution method for HIV positives and air displacement plethysmography for HIV negative adults). As untreated HIV infection leads to loss of lean body mass, it can result in reduced serum creatinine pool and the serum creatinine level may lie within normal range despite decreased GFR and impaired renal function that HIV patients may have due to HIV infection.

HIVAN is predominantly present in people of African ancestry [21,22], and studies have associated the high prevalence of HIVAN in African descent with the presence of apolipoprotein L1 (APOL11) gene in Africans [23–25]. However, Ethiopian studies showed absence of HIVAN in Ethiopian HIV patients [26,27]. We found higher serum creatinine in WHO stage IV compared to WHO stage I HIV positive adults which may be due to the severity of infections in WHO stage IV diseases resulting in acute kidney injury in the patients. However, we found no difference in eGFR between HIV positive and negative persons using all GFR estimating equations except the CG equation which showed borderline significantly higher eGFR in HIV negative persons. This may be due to the small number of patients with advanced clinical disease (WHO stage IV) recruited in our study or due to less susceptibility of Ethiopians to HIV associated kidney injury due to absence of APOL1 gene [23–25,27]. However, this may also be due to the overestimation of eGFR in HIV patients with low body surface area as discussed below.

Though eGFR equations account for some of the variability in muscle mass as it includes age, sex and weight; most of the equations were developed in western settings where individuals had higher body weight and muscle mass [7,8]. Hence, the equations may be inaccurate and tend to overestimate eGFR when applied on individuals with very low muscle mass. Moreover, most of the equations are developed in settings where individuals had high body surface area (>1.73 m^2^) while our study population had smaller body surface area which also may contribute to the overestimation of eGFR as it is described as ml/min/1.73 m^2^. Our data also showed that the CG equation performed better when body surface area adjustment was removed.

There was high correlation between the various eGFR equations and relatively low correlation was seen when the equations are compared to 24-hr creatinine clearance. CKD-EPI without ethnic correction had the best overall performance, whereas CKD-EPI and MDRD equations showed highest bias and lowest accuracy when ethnic factor correction for black was used. Previous studies that were done in other parts of Africa also showed similar findings [28,29]. In addition, the African-Americans who were included in the study during the development of eGFR equations, where the coefficient for blacks were derived from, had higher weight and body surface area compared to our study population [8,30]. Moreover there are dietary differences between the African-Americans and our study population. Therefore, the difference in muscle mass and diet affect the creatinine pool and may result in overestimation of eGFR. For all equations, the accuracy decreased and the bias increased for participants with creatinine clearance of < 90 mL/min/1.73 m^2^. This shows that eGFR equations overestimate GFR especially in patients with low GFR and may shadow the real prevalence of chronic kidney disease in HIV positive adults.

The study looked at the renal function in HIV positive and negative with detail anthropometric and body composition data. However, the study had some limitations that we had not used gold standard GFR measurement for comparisons and also only single measurement of 24-hour urine collection urine was used to determine 24-hr creatinine clearance in a relatively small sample of HIV positive adults who volunteered.

## Conclusion

This cohort of Ethiopian HIV adults eligible for ART had good kidney function at the initiation of ART. Among the most commonly applied eGFR equations, the CKD-EPI without ethnic correction had the best overall performance. However, all eGFR equations overestimated 24-hr creatinine clearance in this study population. This may result in underestimation of renal impairment in African HIV patients and also may mask early identification of renal disease. Creatinine based eGFR equations that account for low muscle mass and body surface area are needed. Long term follow-up studies of renal function changes with ART initiation are warranted.

## Supporting information

S1 Table Dataset. (CSV)
